# Engineering ovarian tissue via biofabrication and 3D bioprinting: Challenges and emerging perspectives

**DOI:** 10.1002/btm2.70088

**Published:** 2025-11-12

**Authors:** Chieh En Hsu, Mario Mazza, Giordano Perini, Antonio Minopoli, Valeria Ferrara, Caterina Perfili, Giulia Artemi, Alberto Augello, Valentina Palmieri, Claudia Marchetti, Raffaella Ergasti, Camilla Nero, Marco De Spirito, Massimiliano Papi

**Affiliations:** ^1^ Faculty of Medicine and Surgery Università Cattolica del Sacro Cuore Rome Italy; ^2^ Cardiology Department School of Medicine and Surgery Milano‐Bicocca University Milan Italy; ^3^ Dipartimento di Neuroscienze Università Cattolica del Sacro Cuore Rome Italy; ^4^ 3D Bioprinting Facility Department of Woman, Child and Public Health Fondazione Policlinico Universitario “A. Gemelli” IRCSS Rome Italy; ^5^ Institute for Complex Systems Consiglio Nazionale delle Ricerche Rome Italy

**Keywords:** biofabrication, bioprinting, ovary, reproductive system, tissue engineering

## Abstract

The development of bioprosthetic ovaries using advanced biofabrication and 3D bioprinting technologies has achieved significant attention in recent years. This work begins with an analysis of ovarian anatomy and physiology, emphasizing the critical structural and functional components that must be replicated for an effective engineered in vitro model. It further outlines the principles and capabilities of 3D bioprinting, with a focus on the customization of printing modalities and bioinks to closely mimic native ovarian tissue. Given the ovary's dual functions in gametogenesis and endocrine signaling, attention is given to how engineered constructs can be designed to restore hormonal homeostasis through the precise spatial arrangement and biological activity of embedded cells. Finally, the technical challenges and ethical considerations associated with translating bioprinted ovarian tissues into clinical applications are discussed.

## Graphical abstract

(a) Loss of ovarian function reduces sex hormone levels, affecting the whole body. (b) A potential solution involves isolating theca and granulosa cells from follicles for 3D bioprinting. (c) Strategies include bioprinting these cells in hydrogels or seeding them onto pre‐printed ovarian scaffolds. (d) The bioprinted ovaries can be implanted to restore hormonal balance.

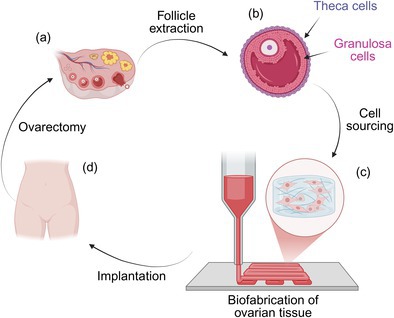

**Abbreviations**
3D: three‐dimensional
BMSC: bone marrow‐derived mesenchymal stem cells
BRCA: breast cancer gene
CD‐1: cluster of differentiation‐1
DYN: dynein or dynamine
ER‐α: estrogen receptor alpha
FSHR: follicle‐stimulating hormone receptor
GDF‐9: growth differentiation factor‐9
GelMA: gelatine‐methacrylate
GnRH: gonadotropin‐releasing hormone
KISS: not present in the text
MII: metaphase II
NKB: neurokinin B
PAS: periodic acid schiff
PLO: poly‐L‐ornithine
POCs: polycystic ovary syndrome
UV: ultraviolet
UV‐Vis: ultraviolet‐visible

**Translational Impact Statement**

Premature ovarian failure due to surgery, cancer therapy, or genetic risk reduction leads to infertility and systemic health decline, and current treatments such as hormone replacement or tissue transplantation offer only partial solutions. Advances in 3D bioprinting and biofabrication now enable the creation of ovarian constructs that replicate both hormone production and follicle development, showing promising results in preclinical models. These technologies could transform care by restoring fertility and endocrine health while also providing platforms for disease modeling and drug testing. Successful translation will depend on overcoming challenges of long‐term function, vascularization, regulation, and ethics, but the potential impact on women's health is profound.

## 1 INTRODUCTION

The ovary plays a pivotal role in female health, functioning not only as the site of gametogenesis but also as a central endocrine organ regulating systemic homeostasis through the production of sex steroid hormones. These hormones are fundamental in maintaining cardiovascular integrity, bone density, neurocognitive performance, metabolic balance, and overall quality of life throughout the reproductive lifespan and beyond. Disruption of ovarian endocrine function, whether physiological (menopause) or iatrogenic (surgical removal), therefore has far‐reaching multisystemic consequences. Oophorectomy, the surgical removal of one or both ovaries, is a common gynecological procedure indicated for a variety of conditions, including primary benign or malignant ovarian tumors and symptomatic masses that may cause pain or increase the risk of complications such as ovarian torsion. When performed with bilateral salpingectomy, it also serves as a preventive strategy in individuals with hereditary cancer syndromes, such as BRCA1 or BRCA2 pathogenic variant carriers, who are at increased risk for breast and ovarian cancer.^1^, ^2^ Risk‐reducing bilateral salpingo‐oophorectomy (RRBSO) is typically recommended between the ages of 40 and 45, leading to abrupt and premature surgical menopause when performed in premenopausal women. This results in a sudden and significant decline in circulating sex steroid hormones, particularly estrogens, with wide‐ranging systemic effects.^3^, ^4^

Endogenous estrogens exert critical protective roles,^5^, ^6^, ^7^ consequently, premenopausal bilateral oophorectomy is associated with increased risks of cardiovascular disease (CVD), decreased bone mineral density (BMD), heightened fracture risk, cognitive impairment, and increased all‐cause and disease‐specific mortality.^8^ Additionally, surgical menopause often leads to significant impairments in quality of life due to vasomotor instability, urogenital atrophy, sexual dysfunction, and psychosocial disturbances.

Hormone replacement therapy (HRT) remains the primary clinical strategy to mitigate the adverse sequelae of premature estrogen deprivation, and is suggested until the age of natural menopause in healthy women, with no contraindications, including those BRCA 1/2 mutation carriers undergoing RRBSO.^9^, ^10^, ^11^ Despite its established benefits—including amelioration of vasomotor symptoms, preservation of BMD, and improved neuropsychological well‐being—HRT uptake is hindered by concerns regarding individual contraindications, and its efficacy in fully restoring the complex endocrine and paracrine signaling of the ovary remains limited.^9^, ^10^, ^11^

Alternative strategies such as ovarian tissue cryopreservation (OTC) and subsequent autologous transplantation (OTT) offer potential avenues for hormonal restoration, but are constrained by technical complexity and the theoretical risk of reintroducing malignant cells, particularly in BRCA mutation carriers.^12^, ^13^ In this context, regenerative medicine and biofabrication technologies are emerging as transformative paradigms for ovarian function restoration.

Modern 3D bioprinting technologies offer a promising alternative for restoring ovarian endocrine function, particularly in women who undergo premature bilateral oophorectomy.^14^ Bioprinting approaches involve the use of preserved ovary tissue from the patient, which can be transplanted into a meticulously engineered environment that mimics the native tissue microarchitecture, supporting both structural integration and hormonal function while maintaining immunological compatibility. At the core of this innovation lies bioprinting—a cutting‐edge technique that enables the precise layer‐by‐layer deposition of biomaterials and living cells to fabricate tissue‐like structures. Unlike conventional tissue engineering, which relies on static scaffolds, bioprinting allows for the dynamic design of three‐dimensional constructs that closely replicate the spatial complexity, mechanical properties, and cellular heterogeneity of natural organs.^15^, ^16^, ^17^ This is particularly critical in the case of the ovary, where the coordination between multiple cell types—including oocytes, granulosa cells, and theca cells—is essential for regulated hormone production and follicular development. In some cases, the source of these cells may be autologous, while in others, they may be derived from induced pluripotent stem cells (iPSCs), offering an alternative pathway for restoring function in women who lack viable ovarian tissue.^18^

In this Review, we explore the state of the art in the development of bioprosthetic ovaries using modern biofabrication strategies and 3D bioprinting techniques. We begin with an overview of ovarian anatomy and physiology, emphasizing the key features that must be recreated to achieve functional restoration. We then present the principles and capabilities of 3D bioprinting technologies, highlighting how different printing modalities and bioinks are tailored to mimic native ovarian tissue. Given the ovary's dual role in both gametogenesis and endocrine signaling, we examine how engineered constructs can be designed to restore hormonal homeostasis through the spatial arrangement and biological activity of embedded cells. Finally, we address the technical limitations and ethical considerations that accompany the translation of bioprinted ovarian tissues into clinical therapies.

## 2 OVARIAN STRUCTURE FUNCTION AND HORMONES

The ovary is a structurally dynamic organ which alters its microscopic organization throughout a woman's life. The structural changes influence its endocrine function, through an alteration of paracrine status, resulting in modification of folliculogenesis and oocyte development.^19^, ^20^ The dimensions of ovaries tend to increase during a woman's lifespan^21^, ^22^ until reaching the reproductively mature size of roughly 4 × 2 × 3 cm. During pregnancy, ovaries double in volume, but after menopause, their size decreases significantly.

Ovarian complex histology and anatomical connections with other parts of the female reproductive system are essential for its function. In a non‐pregnant adult, the ovaries are suspended in the pelvic cavity by the mesovarium. This area is called the ovarian fossa, which is bordered laterally by the parietal peritoneum, posteriorly by retroperitoneal structures and medially by the uterus and its vessels in the broad ligament. The ovaries receive their blood supply through the mesovarium, with ovarian vessels passing through the hilum amidst the nerves. Regarding veins, they emerge from the pampiniform plexus and traverse as two distinct vessels in the mesovarium and suspensory ligament. These vessels subsequently amalgamate into a singular vein before draining into either the inferior vena cava. Lymphatic drainage follows veins to para‐aortic nodes situated near renal arteries.^23^

**FIGURE 1:**
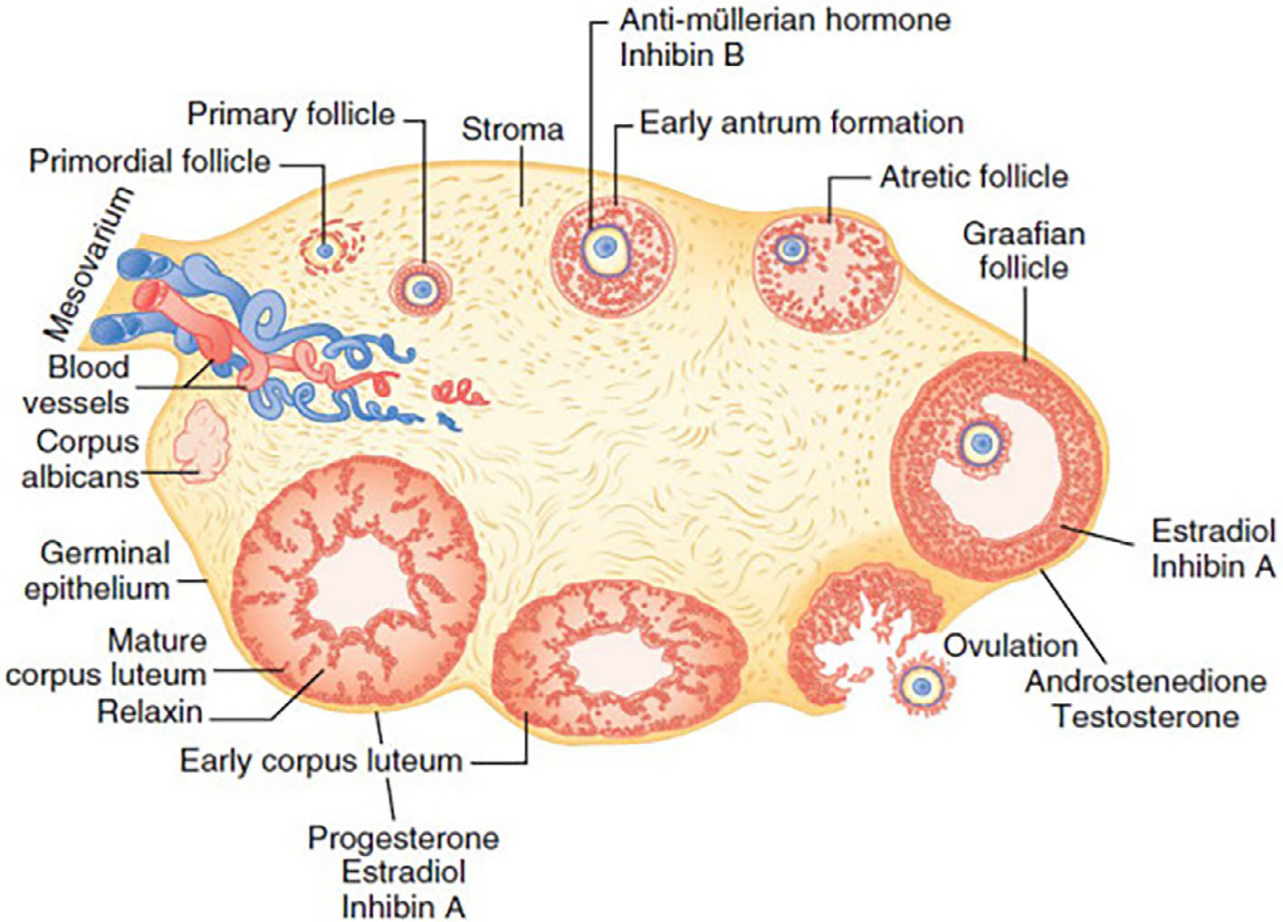
The follicular cycle of the human ovary and main secretory products. Schematic representation of the human follicular ovarian cycle and the main products' secretion. The image shows the progression from primordial follicles to Graafian follicles, ovulation, and corpus luteum formation. Supporting structures like the stroma, blood vessels, and mesovarium are also depicted. Reproduced from Reference [24] © 2019 Elsevier Inc.

The ovarian microscopic structure can be subdivided into two distinct regions, the cortex in the periphery, containing the ovarian follicles, and the medulla in the central portion, receiving vessels and nerves at the hilum (Figure 1). The boundary between these regions is indistinct. Before puberty, the ovary consists mainly of the cortex, which takes up approximately 35% of its volume. Meanwhile, the medulla and interstitial cells occupy 20% and up to 45% of the volume, respectively. As puberty sets in, the cortex takes on a more prominent role, enveloping the medulla except for the hilum.

The cortex contains ovarian follicles of various sizes, strictly linked with the development stage of oocytes, embedded in a dense stroma composed of thin collagen fibers and fusiform fibroblast‐like cells, rich in lipid droplets and arranged in whorls. These cells give rise to the thecal layers of mature ovarian follicles. The distribution and frequency of primordial follicles containing oocytes in the ovaries is not uniform, and the number of primordial follicles containing oocytes varies based on patients' ages. The quantity of follicles decreases with age and can vary between the two ovaries, from an equal distribution to a significant disparity.^25^

During puberty, small groups of follicles undergo a cyclic process of growth and maturation; only one oocyte for each menstrual cycle, defined as dominant, reaches full maturity, for a total of roughly 400 mature oocytes during the reproductive lifespan.^26^

**FIGURE 2:**
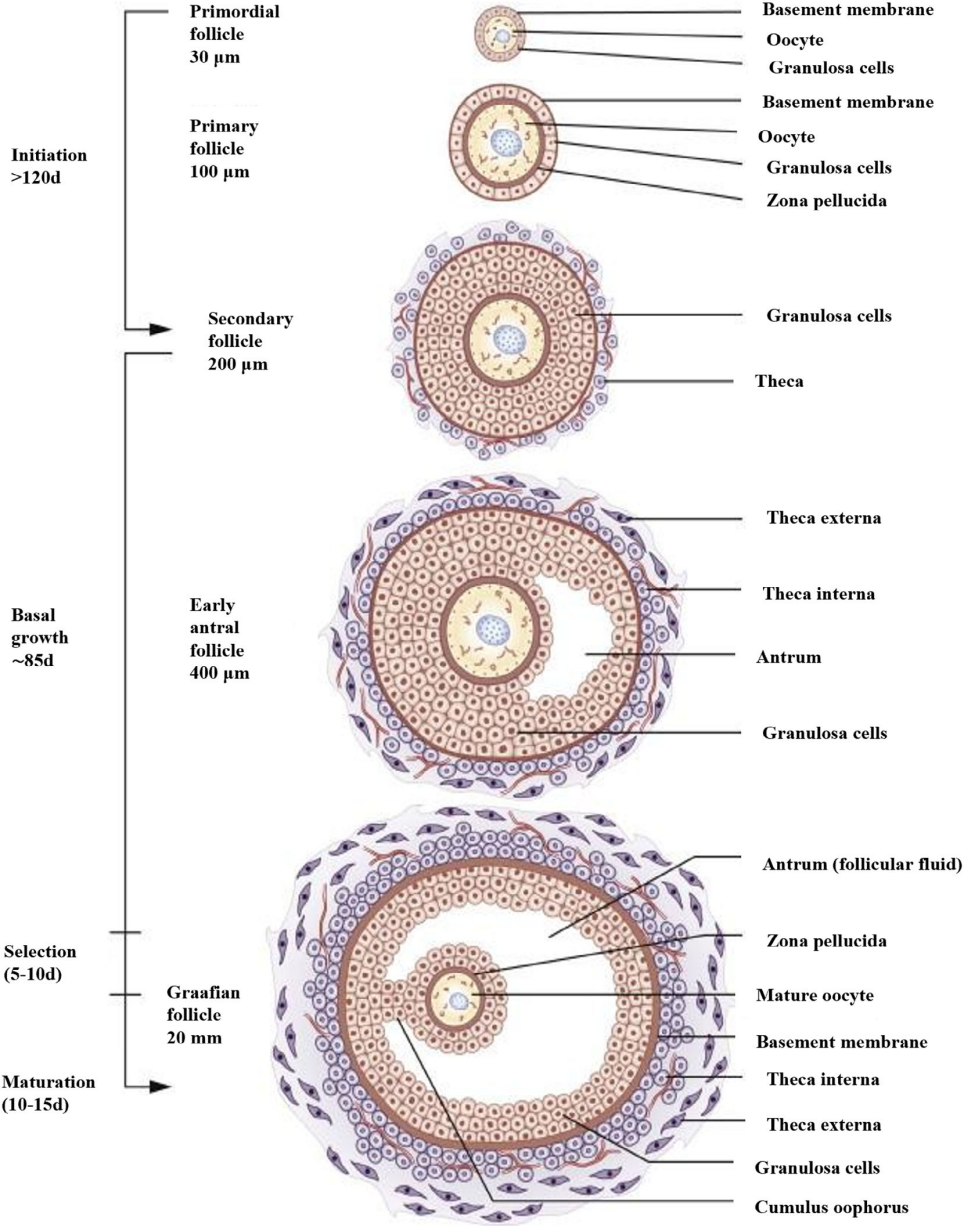
Illustration of human folliculogenesis showing the morphological and structural changes from the primordial to the Graafian follicle stage. The process is divided into phases—initiation, basal growth, selection, and maturation. Each follicular stage is characterized by increasing size and complexity, including the development of the zona pellucida, formation of granulosa and theca layers, antrum formation, and eventual appearance of the cumulus oophorus surrounding the mature oocyte. The figure highlights critical anatomical components such as the basement membrane, theca interna and externa, and follicular fluid‐filled antrum. Reproduced from Reference [28] © 2015 Elsevier Inc.

Histologically, there can be three basic types of ovarian follicles, according to developmental state: primordial, growing and mature follicles. The primordial follicle is the earliest stage of follicular development, and its growth is gonadotropin independent. It emerges in the third month of fetal life and can be found in the ovary's mature stroma of the cortex just below the tunica albuginea.^27^ As egg follicles grow, they become primary or secondary (Figure 2). As the secondary follicles grow, the stratum granulosum also enlarges, forming a thickened mound known as the cumulus oophorus, projecting into the antrum. The oocyte surrounding cells are equipped with microvilli that penetrate the zona pellucida. These microvilli increase in number as the Luteinizing hormone (LH) receptor increases, and after ovulation, they will become the corona radiata. Only one tertiary follicle usually develops during a menstrual cycle, while others become atretic. The mature follicle, called Graafian follicle, has a 10 mm diameter. The granulosa layer mitotic activity decreases, it becomes thinner, and the antrum decreases in size. The cumulus cells are loosened and corona radiata develop a single layer of cells. Thecal layers become more prominent, and lipid droplets appear in the cytoplasm of theca interna cells and start producing steroids. The follicle extends to the full thickness of the cortex and causes a bulge on the ovarian surfaces. The primary oocyte completes its first meiotic division to produce the secondary oocyte and the first polar body. The secondary oocyte begins its second meiotic division but pauses at metaphase until fertilization takes place. The point of contact (the stigma) with the tough tunica albuginea and the ovarian surface epithelium is eroded until the follicle ruptures and releases the oocyte into the peritoneal cavity for capture by the fimbria and actively transported by the ciliated cells lining the uterine tube. If fertilization does not occur, the oocyte deteriorates after 24–48 h. The process that causes the follicle to rupture involves several factors, such as the accumulation of fluid and pressure within the follicle, the enzymatic breakdown of follicular tissue by plasminogen, the deposition of glycosaminoglycans (GAG) between the cumulus and granulosa cells under hormonal direction, and the contraction of the smooth muscle layer in the theca externa, which is triggered by prostaglandins. Ultimately, the blood flow in the stigma region ceases just before ovulation. Following ovulation, the basal lamina breaks down and the follicular wall composed of granulosa and thecal cells collapses and forms the corpus luteum. The capillaries in the theca interna bleed into the follicular lumen leading to the corpus hemorrhagicum. The cavity is then invaded by connective tissue from the stroma, and the granulosa and theca cells undergo luteinization, which involves changes in morphology, increased size, and the accumulation of lipid droplets that test positive for lipochrome.^29^

Granulosa cells develop during primordial follicle maturation, in which follicle cells become cuboidal and later undergo rapid mitotic proliferation, resulting in the formation of the membrana granulosa and the development of the late primary follicles. The granulosa cells are separated from the stroma by the basal lamina. They possess gap junctions but not tight junctions, and cytoplasmic processes radially inwards; these contact and communicate with oolemm and oocyte microvilli in the perivitelline space with gap junctions.^30^ Moreover, they release cyclic guanosine monophosphate (cGMP) to inhibit cyclic adenosine monophosphate (cAMP) hydrolysis by phosphodiesterase 3A (PDEA3) and prevent meiotic progression. Consequently, nutrients and small informational macromolecules need to move from the blood into the follicular fluid to ensure the normal development of the ovum.

During the antral stage, granulosa cells produce hyaluronan‐rich fluid, creating a cavity called the antrum. The liquor folliculi also contains growth factors (GFs), steroid hormones secreted by the granulosa cells and C‐type natriuretic peptide (CNP) in indirect proportion to the amount of liquid. It is one of the factors maintaining oocytes in meiotic arrest. Among the granulosa cells, there are PAS‐positive materials known as Call‐Exner bodies, which are composed of hyaluronan and proteoglycans. After luteinization, they originate granulosa lutein cells, large, centrally located cells constituting 80% of the corpus luteum which synthesize estrogens, progesterone from aromatization of androstenedione synthesized by theca lutein cells, and inhibin.

Thecal cells develop from the stromal fibroblast‐like cells into spindle‐shaped cells arranged in a sheath‐like formation, located externally to the basal lamina in two distinct layers. The inner layer is known as the theca interna, a highly vascularized layer comprised of cuboidal secretory and steroid‐producing cells that contain LH receptors. These cells synthesize and secrete androgenous in response to LH stimulation. Additionally, fibroblasts, collagen bundles, and a network of small vessels are present in this layer. The outer layer is known as the theca externa, which consists of smooth muscle cells and collagen fibers. Although the layers are not clearly defined from one another or the surrounding stroma, they work in tandem to support ovarian function. The thick basal lamina distinguishes the avascular granulosa layer and the rich‐in‐vessel theca interna one, restricting the entry of leukocytes and high‐molecular‐weight substances like low‐density lipoproteins (LDL) into the follicle. In the follicles mature, the theca interna becomes more prominent and its cells more rounded and typical of steroid‐secreting endocrine cells. They produce androstenedione, from which the granulosa cells derive.

Following the process of luteinization, the theca lutein cells are produced. These cells are smaller and situated on the periphery, accounting for 20% of the corpus luteum. They carry out the important function of secreting both androgens and progesterone. Additionally, the blood and lymphatic vessels of the theca interna grow into the granulosa layer, creating a robust vascular network that secretes progesterone and estrogens.

**FIGURE 3:**
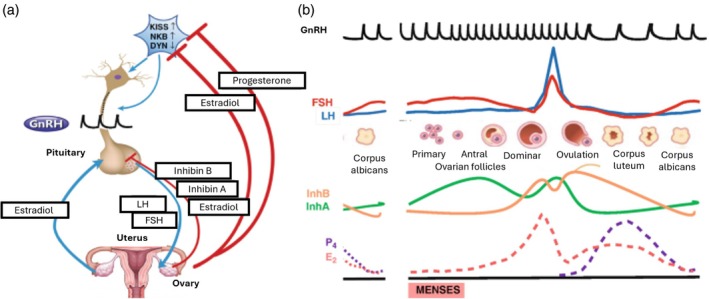
The hypothalamic–pituitary‐ovarian axis during the female reproductive cycle (a) Coordinated hormonal feedback between the hypothalamus, pituitary, ovaries and uterus (b) The regulation of the menstrual cycle is governed by dynamic feedback mechanisms between the ovary, hypothalamus, and pituitary: (i) the rise in follicle‐stimulating hormone (FSH) required to recruit a new cohort of follicles at the transition between cycles, which occurs due to the withdrawal of negative feedback from declining ovarian steroid and peptide hormones following corpus luteum regression; and (ii) the subsequent suppression of FSH by estradiol and inhibins secreted by the developing follicles, which prevents the maturation of multiple follicles. In normal cycles, a positive feedback loop driven by estradiol at the level of the pituitary triggers the pre‐ovulatory surge. Reproduced from References [31, 32] © Springer and The Author(s) respectively. LH, Luteinizing hormone.

The ovarian gland plays an active role in initiating and maintaining reproductive cyclicity and controls the very function of the hypothalamic–pituitary–ovarian axis, as shown in the Figure 3.^31^, ^33^

Gonadotropin‐releasing hormone (GnRH) a hormone produced by the hypothalamus controls the release of LH and follicle‐stimulating hormone (FSH) by the pituitary gland. These hormones are necessary for the growth and maturation of ovarian follicles, ovulation, and the regulation of the menstrual cycle. In the ovary, LH and FSH act on different types of cells, each playing a crucial role in regulating ovarian function, particularly the maturation of follicles and the production of sex hormones. FSH primarily acts on the granulosa cells which are involved in the growth and maturation of the developing follicles that support the oocytes. FSH induces granulosa cells to convert androgens into estrogens (estradiol [E2]) through the enzyme aromatase. This estrogen production is essential for follicle development and the regulation of the menstrual cycle. LH primarily acts on the theca cells which surround the follicles, and LH stimulates them to produce androgens (such as androstenedione and testosterone), which are later converted into estrogens by the granulosa cells under the influence of FSH, as previously anticipated. Additionally, during the ovulation phase, LH also acts on the granulosa cells of the dominant follicle triggering ovulation and after luteinization, the transformation of the remaining follicle into the corpus luteum, which secretes progesterone, crucial for maintaining the uterine lining and preparing it for a potential pregnancy.^34^ E2 is an 18‐carbon steroid, while progesterone is a 21‐carbon steroid.^35^

The menstrual cycle comprises two distinct phases: the follicular phase and the luteal phase. The follicular phase commences on the first day of menstruation and continues until ovulation, typically spanning 10–16 days. It is marked by folliculogenesis, during which serum E2 levels escalate alongside follicle growth and granulosa cell proliferation. In contrast, the luteal phase follows ovulation and typically spans 14 days in most women. This phase is characterized by the formation of the corpus luteum, which primarily secretes progesterone.^36^ In the initiation of the menstrual cycle, E2 continues to rise, leading to the endometrium proliferation and reaching a peak in ovulation. By responding to the positive feedback of the hypothalamic–pituitary axis, E2 reaches a blood concentration of 200 pg/mL for approximately 50 h. Progesterone, on the other hand, peaks 1 week after ovulation, triggering the conversion of the endometrium in preparation for implantation. If the pregnancy does not take place, the progesterone will be lost subsequently and result in the sloughing of the endometrium, initiating a new menstrual cycle.^35^, ^37^ In addition, ovarian peptide hormones also play a role in the menstrual cycle by modulating central nervous system hormone release. They are inhibin, activin, and anti‐Müllerian hormone (AMH), and all of them are secreted by granulosa cells and belong to the transforming growth factor‐beta superfamily (TGF‐beta) of ligands.^22^ Inhibin is a polypeptide that has two forms: inhibin A is predominantly secreted in the luteal phase, while inhibin B is predominantly secreted in the follicular phase.^35^ Inhibin synthesis is stimulated by FSH but diminished by hypophysectomy and progesterone.^38^ Activin enhances FSH secretion but can be counteracted by inhibin and follistatin. Additionally, AMH is produced by granulosa cells in pre‐antral follicles, promoting follicle growth and serving as a marker of their quantity. AMH levels also serve as an indicator of ovarian reserve, potentially aiding in the diagnosis of infertility through blood testing.^35^

## 3 BIOFABRICATION AND 3D PRINTING TECHNOLOGIES IN OVARIAN ENGINEERING

Given the ovary's intricate organization and multifaceted physiological roles—including folliculogenesis, hormone secretion, and cyclic remodeling—engineering a functional ovarian substitute poses an elaborate challenge since it requires the replication of both its structural complexity and dynamic behavior. Traditional tissue engineering approaches have struggled to recreate the heterogeneous cell populations, spatial organization, and responsive extracellular matrix (ECM) that characterize native ovarian tissue as well as reliable ovarian cancer models. These limitations have catalyzed interest in more advanced fabrication techniques capable of recapitulating the ovary's architecture and function with higher precision. Among these, 3D bioprinting has emerged as a powerful platform, offering new opportunities to address the biological and mechanical demands of engineered ovarian constructs.

Development of ovary scaffolds using state‐of‐the‐art 3D bioprinting technologies would constitute a pioneering advancement in tissue engineering, particularly for addressing reproductive health challenges such as infertility. By replicating the complexity of ovarian tissues, these scaffolds aim to support follicle growth and restore hormonal balance in patients who have lost ovarian function due to medical conditions such as cancer or genetic disorders, thereby offering new avenues for clinical applications and patient care.^39^, ^40^

Bioprinting technologies, including extrusion‐based and inkjet, provide a fine control over scaffold design, enabling the precise layering of bioinks containing living cells and biomaterials. This customization is essential for creating an optimal microenvironment that fosters cellular growth and functionality. Recent developments have demonstrated the suitability of various materials, such as decellularized extracellular matrices (dECM) and hydrogels, in constructing scaffolds that closely mimic the natural ovary architecture, thus enhancing the prospects for successful integration and tissue regeneration in vivo.^41^, ^42^, ^43^

Despite the promising advancements, the development of bioprosthetic ovaries and associated scaffolds faces several challenges, including optimizing scaffold structural features, ensuring cell viability, and addressing ethical and regulatory concerns surrounding their clinical use. Additionally, long‐term functionality and biocompatibility of these scaffolds are crucial for successful clinical translation, which may offer safer alternatives to traditional ovarian tissue transplantation.^44^, ^45^, ^46^, ^47^, ^48^, ^49^, ^50^, ^51^

### 3.1 3D bioprinting approaches

The choice of bioprinting strategy is a critical determinant in the successful engineering of ovarian constructs. Beyond the selection of an appropriate bioink, the printing modality itself governs key aspects such as spatial resolution, achievable construct geometry, cell viability, and the capacity to reproduce both the mechanical profile and microenvironmental cues of native ovarian tissue. Different bioprinting platforms operate through distinct physical principles, each offering specific advantages and limitations in terms of structural precision, biological compatibility, and scalability.

**FIGURE 4:**
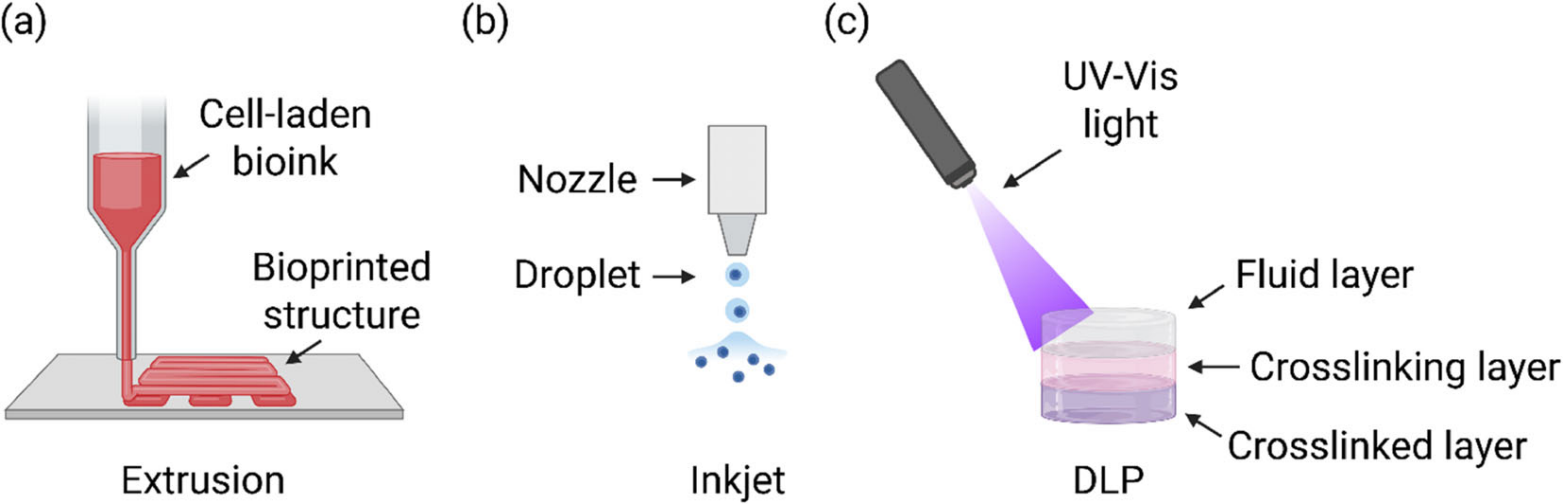
Scheme of several bioprinting strategies (a) Bioink is extruded through either pneumatic pressure or controlled volume pump and crosslinked through either thermal or chemical processes, (b) Bioink droplets are ejected through either thermal or piezoelectric actuation and crosslinked through either thermal or chemical processes, (c) Bioink is crosslinked layer‐by‐layer using UV or visible light with sub‐micrometer resolution. DLP, digital light processing.

Extrusion bioprinting is by far the most widely used technique for fabricating ovarian constructs, mainly because of its versatility and its capacity to process highly viscous bioinks.^52^ In this method, a continuous filament of material is deposited through a nozzle under pneumatic, piston‐driven, or screw‐assisted pressure (Figure 4a). This allows the incorporation of high cell densities into bioinks or hydrogels derived from decellularized ECM, which are often necessary to mimic the dense stromal environment of the ovarian cortex.^40^ Extrusion‐based printing excels in producing structures with defined pore architectures that can provide both mechanical stability and permeability for nutrient diffusion. However, the relatively high shear stress generated during extrusion can negatively affect the viability of sensitive primary cells or immature oocytes.^53^ Balancing viscosity, extrusion pressure, and nozzle diameter is therefore critical to preserve biological function while maintaining structural integrity.

Inkjet, or drop‐on‐demand, bioprinting uses either thermal or piezoelectric actuation to eject precise droplets of bioink onto a substrate in a highly controlled pattern.^54^ This enables high‐resolution placement of multiple cell types, offering the possibility to engineer microenvironments with spatial gradients of GFs or extracellular cues (Figure 4b). Its low cost, scalability, and compatibility with standard cell culture media make it attractive for high‐throughput applications. Nonetheless, because the process requires low‐viscosity bioinks, the resulting constructs typically lack the mechanical strength needed for load‐bearing or long‐term implantation.^55^ In the context of ovarian tissue engineering, inkjet printing may be particularly valuable for positioning small cell aggregates (such as granulosa cells, theca cells, or early‐stage follicles) within a preformed scaffold, rather than producing the entire structural framework.

Digital light processing (DLP) and stereolithography (SLA) rely on the selective exposure of a photosensitive bioink to patterned light, crosslinking the material layer by layer (Figure 4c). These methods can achieve high resolution, enabling the fabrication of constructs with intricate internal geometries that closely replicate native tissue microenvironments. Light‐based printing is especially well suited for creating microchannel networks that could support vascularization or for replicating the compartmentalization of the ovarian cortex and medulla.^56^ However, the choice of bioink is constrained by the need for photocrosslinkable chemistry and cytocompatible photoinitiators, and care must be taken to avoid phototoxic effects. For reproductive tissues, where long‐term hormonal function is essential, ensuring that the photo‐crosslinked network supports both cell viability and dynamic remodeling is a key consideration.^57^

Each bioprinting approach offers a different balance between resolution, mechanical fidelity, and biological compatibility. Extrusion‐based techniques currently dominate in ovarian tissue engineering due to their ability to handle viscous bioinks that approximate the stiffness of the ovarian cortex while allowing compartmentalized cell deposition.^52^ Inkjet and laser‐assisted methods, while less suited for building bulk mechanical structures, are highly valuable for patterning cells with high precision and could be integrated with extrusion printing to enhance microarchitectural complexity. Light‐based printing methods hold significant promise for replicating the detailed geometry of the ovarian microenvironment, provided that bioink chemistries compatible with reproductive cell survival are employed. Ultimately, hybrid strategies that combine the structural robustness of extrusion with the resolution of inkjet or DLP may offer the most effective route toward constructing functional, clinically relevant ovarian substitutes.

### 3.2 Decellularized ovaries and bioinks

The selection of appropriate biomaterials is a fundamental requirement for the successful bioengineering of ovarian scaffolds, and the biomechanical properties of these materials should ideally align with the specific needs.

Many hydrogels have been investigated for developing ovarian tissue, such as alginate, collagen, fibrin, Matrigel, agar, laminin, and GelMA, each possessing distinct properties that can enhance the survival of transplanted oocytes.^58^ These hydrogels are able to mimic the 3D architecture of ovarian follicles and the biochemical microenvironment that maintains communication with stromal cells. Alternatively, decellularized ovaries offer a natural ECM scaffold that preserves the native structure and biochemical cues of the ovarian tissue. This allows for better mimicry of the ovarian microenvironment, supporting follicle attachment, growth, and function. Decellularized ovaries retain structural proteins, GFs, and signaling molecules essential for tissue regeneration, which can enhance the survival and maturation of transplanted follicles.^59^ However, they may require more complex preparation and have limited availability compared to synthetic or bioengineered alternatives like hydrogels. Decellularized ovarian tissue serves as a natural bioscaffold, retaining a complex ECM architecture enriched with key biomolecules such as collagens, GAGs, and glycoproteins. These components are crucial for supporting folliculogenesis and maintaining the physiological functions of ovarian tissue. Various decellularization protocols have been employed to process mammalian ovaries, utilizing combinations of physical disruption, chemical detergents, and enzymatic treatments to effectively remove cellular components while preserving the structural and biochemical integrity of the ECM.^60^, ^61^, ^62^, ^63^, ^64^, ^65^ Hassanpour and colleagues developed a decellularization protocol using 1% sodium lauryl ester sulfate (SLES) followed by DNase I treatment to effectively decellularize human ovarian tissue while preserving the ECM architecture and biochemical composition. The resulting decellularized ovarian scaffolds were subsequently repopulated with primary ovarian cells and implanted in vivo into ovariectomized rats. Post‐transplantation analyses demonstrated cell viability, formation of follicle‐like structures, and restoration of endocrine function, as evidenced by increased serum E2 and progesterone levels.^60^ Other protocols have been used for the decellularization of porcine ovaries, specifically a freeze–thaw cycle followed by sequential incubations in 0.5% sodium dodecyl sulfate (SDS), 1% Triton X‐100, and 2% deoxycholate^61^ and the scaffold was suitable for cell seeding using isolated purified female germline stem cells.^62^ Eivazkhani and colleagues conducted a comparative analysis of two decellularization protocols—SDS and sodium hydroxide (NaOH)—on ovarian tissues from mice, sheep, and humans to assess their suitability for constructing bioengineered ovarian scaffolds. The study demonstrated that NaOH treatment resulted in superior preservation of ECM integrity and supported higher cell metabolic activity compared to SDS. Following decellularization, human ovarian scaffolds treated with NaOH were repopulated with mouse ovarian cells and transplanted in vivo into ovariectomized mice. Post‐implantation evaluations revealed the formation of follicle‐like structures, positive GDF‐9 expression, and enhanced biocompatibility, indicating that the NaOH‐derived scaffold effectively supported cellular survival and partial folliculogenesis.^63^

Artificial tissue should provide structural support during regeneration and degrade at an appropriate pace throughout the menstrual cycle. Additionally, the volumetric changes that occur during folliculogenesis, pregnancy, and hormonal regulation must be considered when designing biomimetic scaffolds. Biomaterials must also possess specific mechanical properties such as elasticity, tensile strength, elongation, and hardness to support organ function. These mechanical properties not only provide necessary support during tissue regeneration but also influence the flow of cellular or tissue fluids and paracrine factors. The ovarian cortex is notably more rigid than the medulla due to variations in the distribution of matrisome proteins.^66^ These differences in tissue rigidity play a crucial role in folliculogenesis, as the process is influenced by the mechanical properties of the ovarian tissue.^67^ Moreover, diverse bioinks can be bioprinted sequentially to form more complex and compartmentalized structures that spontaneously fuse and merge together. Tissue fusion is indeed a self‐assembly process where multiple multicellular structures come into contact and coalesce, a process essential for forming complex structures. Shafiee et al., demonstrated that accelerating the fusion of cellular bioink particles is crucial for achieving tissue functionality.^68^ A higher speed in the maturation of Chinese hamster ovary and fibroblast 3D‐bioprinted structures was indeed obtained through modulated apparent tissue surface tension.^69^

Standard in vitro assays used to evaluate the effects of different components on the growth and maturation of isolated follicles often employ alginate hydrogel due to its ease of gelation, biological inertness, and the ability to modify physical rigidity by adjusting the alginate concentration or varying the calcium crosslinker.^70^ However, alginate has limitations for use in a bioprosthetic ovary aimed at supporting long‐term endocrine and ovulatory functions, as it is not easily remodeled by ovarian cells or infiltrated by blood vessels, which restricts oxygen supply.

Unlike alginate, fibrin is more easily degraded by proteolytic enzymes produced by granulosa and theca cells.^71^, ^72^ Fibrin‐based materials have been used to study follicle growth and survival in various species, such as goats, humans, and rhesus macaques.^73^, ^74^, ^75^ Isolated rhesus macaque follicles encapsulated in fibrin–alginate produced larger follicles, more E2, vascular endothelial growth factor (VEGF), and AMH compared to follicles encapsulated in alginate hydrogels alone.^73^

Other naturally occurring ECM proteins from the ovary, such as hyaluronic acid (HA)‐alginate, were found to produce more oocytes and significantly higher E2 levels compared to both alginate and fibrin hydrogels.^76^ Desai et al.^77^ confirmed these findings, showing that murine follicles encapsulated in HA and Matrigel exhibited higher rates of germinal vesicle breakdown, MII formation, and E2 production compared to controls. A decade later, functional competence was reported, with 82% of oocytes retrieved from follicles matured in HA hydrogels capable of fertilization.^78^

Several studies have demonstrated follicle survival and successful fertility restoration in vivo using aggregated murine follicles or ovarian grafts encapsulated in fibrin or HA‐based matrices, with or without additional GFs.^71^, ^72^, ^79^, ^80^, ^81^, ^82^ Furthermore, research on human ovarian tissue encapsulated in fibrin clots with VEGF and xenografted into mice showed a significant increase in proliferating follicles compared to unencapsulated tissue.^83^

Beyond ECM, Manavella et al. hypothesized that embedding fibrin with proangiogenic adipose tissue‐derived stem cells would enhance revascularization, reducing hypoxia and ischemic injury during tissue transplantation.^79^, ^80^, ^81^ The inclusion of stem cells in these transplants resulted in increased oxygen tension (pO2), greater vessel area and enhanced primordial follicle survival.^71^, ^72^, ^73^, ^74^, ^75^ These findings, with an emphasis on critical microenvironmental factors and tissue revascularization, are fundamental to the eventual development of a bioengineered ovary for human ovarian tissue or follicle transplantation. A comprehensive list of methods and bioinks for ovary bioprinting is reported in Table 1.

**TABLE 1:** Overview of biofabrication strategies and bioinks for ovary engineering.

Bioprinting method	Bioink	Crosslinking method	Cells	Cell density	References
Jetting	Matrigel	Thermal gelation	OVCAR‐5 MRC‐5	1.0–10 × 10^6^ cells/mL	84
Extrusion	Gelatin	EDC/NHS	Follicles	3–4 follicles per scaffold	85
Extrusion	GelMA	Photocuring at 405 nm (GelMA)	COV434 KGN ID8 Primary ovarian cells	2.0 × 10^6^ cells/mL	50
	Alginate	CaCl_2_‐mediated crosslinking			
	GelMA–alginate	Photo‐chemical crosslinking (405 nm, CaCl_2_)			
Extrusion	dECM	CaCl_2_‐mediated crosslinking	Primary ovarian cells	1.0 × 10^6^ cells/mL	86
Extrusion	Alginate Gelatin	CaCl_2_‐mediated crosslinking	SKOV‐3 MeWO	0.5–1.0 × 10^6^ cells/mL	87

Abbreviations: dECM, decellularized extracellular matrices; EDC, *N*‐(3‐dimethylaminopropyl)‐*N*′‐ethylcarbodiimide; NHS, *N*‐hydroxysuccinimide.

**FIGURE 5:**
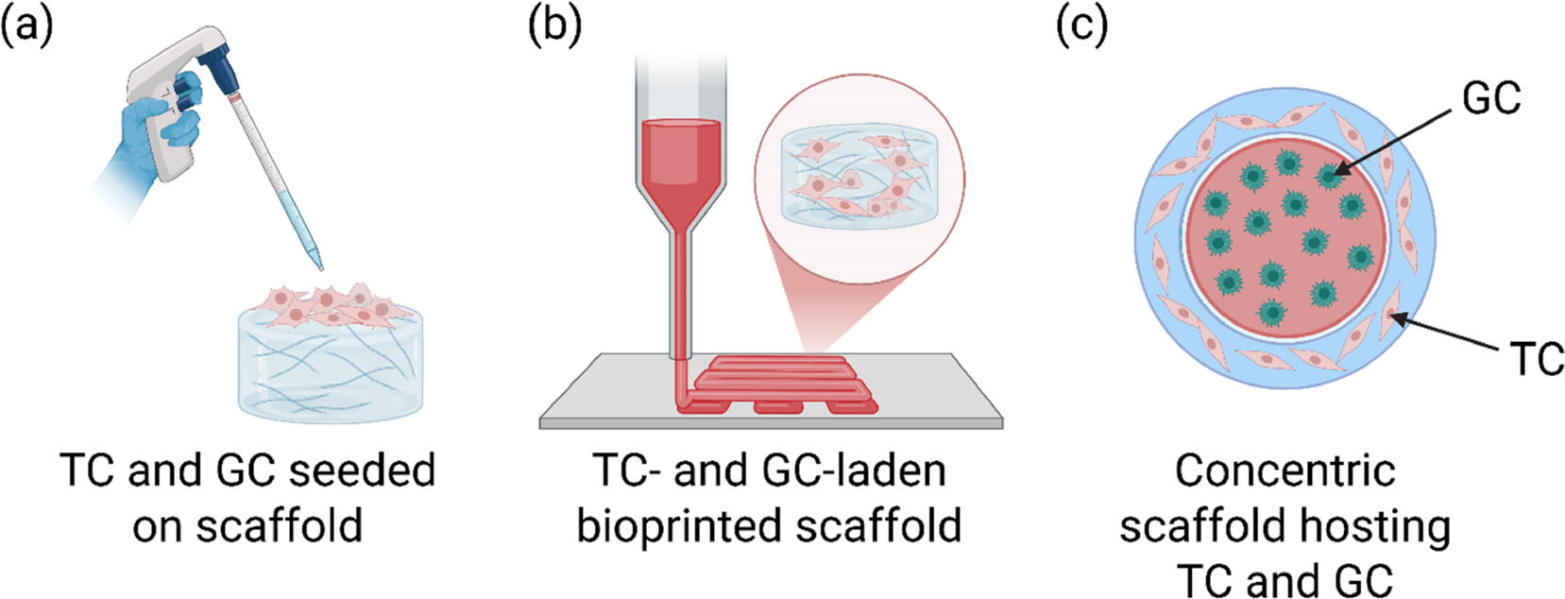
Scheme of the main approaches adopted for ovary biofabrication (a) Theca cells (TC) and granulosa cells (GC) seeded on 3D scaffolds, (b) Bioprinting of TCs and GCs encapsulated in bioinks (e.g., GelMA, alginate, Matrigel), (c) Oocyte‐like architecture hosting GCs and TCs. Created with BioRender.com.

Building upon these biomaterials used to recreate the ovarian microenvironment, diverse bioprinting strategies, particularly extrusion‐ and droplet‐based, can be used in the attempt to reproduce native tissue complexity that results in compatibility from a histological and functional point of view. This includes selecting multiple cell types, scaffold geometries, and microenvironmental features. The transition from material selection to functional tissue fabrication underscores the synergistic relationship between bioink formulation and bioprinting strategy in developing clinically relevant ovarian constructs. Biofabrication of the ovary has currently been performed through different approaches as highlighted in Figure 5: theca and granulosa cells seeded on scaffolds and direct 3D bioprinting of cell‐laden bioinks.

### 3.3 Cell seeding on scaffolds

**FIGURE 6:**
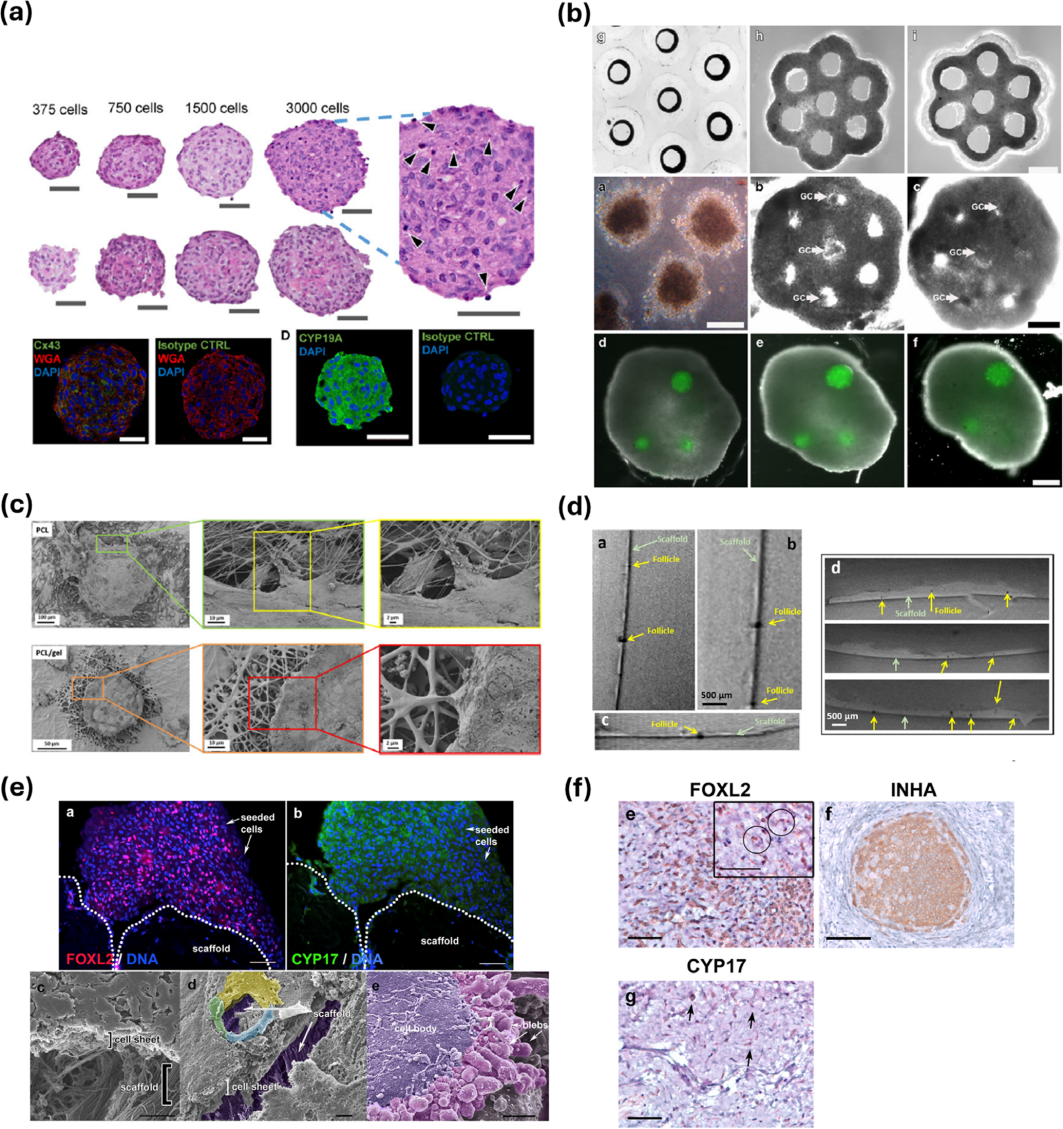
Cell seeding on scaffolds. (a) Granulosa microtissues grown in agarose micromolds showing critical markers of granulosa layers in ovarian follicles through immunostaining. Reproduced from Reference [88], © The Author(s) 2021. (b) Optical images of artificial human ovaries made of theca microtissues and granulosa spheroids. Reproduced from Reference [89], © Springer Science+Business Media, LLC 2010. (c) Scanning electron microscopy of electrospun fibers on which porcine follicles were seeded. Reproduced from Reference [90], © The Author(s) 2019. (d) Magnetic resonance imaging of follicle infiltration inside electrospun fibers. Reproduced from Reference [42], © 2019 The Author(s). (e) Fluorescence images after 48 h show that primary ovarian cells remained mainly on the scaffold surface, whereas a minor fraction migrated through the decellularized medulla scaffold. (f) Representative image from renal graft stained with FOXL2 and alpha‐Inhibin (granulosa cells) and CYP17 (theca cells). Reproduced from Reference [91], © 2015 Elsevier Ltd.

Other biofabrication strategies, different from bioprinting, have been used to resemble ovarian physiology and structure, such as micromolding and electrospinning. Blanche and colleagues developed an agarose micromold to produce 3D granulosa microtissues starting from spheroids.^88^ To create consistent and physiologically relevant microtissues, the authors developed a custom agarose micromolding technique. The authors designed with computer‐aided design (CAD) software a mold tailored to fit the dimensions of a standard 96‐well plate and featured small conical protrusions designed to create equally sized micro‐recesses in the agarose. These conical posts served as negative templates around which the agarose would form miniature wells. A 2% solution of molten agarose was poured into each well. Before agarose solidification, the aluminum mold was inverted and pressed down onto the liquid agarose. As a result, they obtained a hydrogel scaffold containing four well‐defined micro‐recesses per well, each serving as a niche for granulosa spheroids. These spheroids self‐assemble into layered microtissues that closely resembled the avascular granulosa layers in the ovarian follicle. Their 3D structures showed expression of critical markers such as CYP19 (the aromatase enzyme responsible for E2 synthesis) and connexin 43, the protein forming gap junctions that mediate cell‐to‐cell communication in vivo (Figure 6a). Functionally, the microtissues faithfully resembled key features of granulosa cell physiology, particularly the production of E2 from testosterone. Overall, their work introduces a physiologically relevant and versatile model of granulosa microtissues for assessment of hormone synthesis and cell communication.

A similar structure, but with a different purpose was obtained by Krotz et al.^89^ They developed a construct made not only by granulosa cells, but also by theca cells, starting from primary human ovarian cells. Their aim was to produce an artificial ovary to support the full maturation of primordial oocytes, potentially expanding the pool of oocytes available for fertility preservation and improving outcomes for women facing gonadotoxic treatments. They isolated theca and granulosa cells from antral follicles of women undergoing oophorectomy for benign reasons. Both primary cell lines were expanded to form complex 3D structures. Authors used CAD to design a specific negative mold for agarose, by using polydimethylsiloxane (PDMS). After agarose mold formation, theca cells were seeded to form a honeycomb structure. Granulosa cells were cultured separately to form spheroids. These were then successfully inserted into the honeycomb cavities formed by theca cell microtissues (Figure 6b). Another step forward was made by embedding within the theca honeycomb cumulus‐oocyte complexes (COCs), which include an immature oocyte surrounded by granulosa cells. As a result, artificial ovaries maintained cell viability and structural integrity for several days in culture. Importantly, oocytes successfully extruded a polar body, indicating progression to the metaphase II stage of maturation, a critical step for fertilization competence.

Artificial ovaries were also explored by Liverani et al.^90^ Using electrospinning to allow precise control over fiber diameter, porosity, and overall architecture, authors developed poly(ε‐caprolactone) (PCL) and a PCL/gelatin blend. To mimic the human ovarian microenvironment, researchers isolated porcine follicles, capable of reproducing human folliculogenesis dynamics closer than mice. After follicle isolation, they were seeded onto the electrospun scaffolds and cultured for up to 30 days to assess viability, adhesion, morphology, hormone production and follicle infiltration into the porous scaffolds (Figure 6c). Authors observed sustained follicle viability in both scaffolds. However, PCL/gelatin scaffolds outperformed PCL in follicle adhesion and stronger interaction with scaffold fibers. Furthermore, hormone assays revealed that follicles cultured on both scaffolds produced increasing levels of E2 and progesterone over time, indicating active granulosa cell function and follicular development. A critical issue in follicle adhesion is their poor infiltration into electrospun scaffolds due to the dense packing of fibers. To address this point, Raffel and colleagues, from the same research group as Liverani, investigated different techniques to observe follicle infiltration into electrospun PCL and PCL/gelatin scaffolds, having a microporous structure of 300 μm.^42^ Testing standard histological analysis and scanning electron microscopy, they did not manage to properly observe cell infiltration. They therefore suggested another approach: magnetic resonance imaging (MRI). Authors embedded their scaffolds in agarose to avoid movement artifacts and imaged them using a high‐field 7T MRI system. MRI allowed non‐invasive, high‐resolution visualization of both scaffold structure and follicles. Follicles appeared as dark spots (due to lower proton density), clearly distinguishable within the lighter scaffold matrix (Figure 6d). This approach confirmed that follicles not only adhered to the surface but also infiltrated the inner scaffold architecture, especially in the PCL/gelatin group.

Laronda et al.^91^ investigated whether a decellularized ovarian scaffold, recellularized with primary ovarian cells, could restore endocrine function and initiate puberty in ovariectomized mice. Bovine ovaries were decellularized, preserving ECM structure, and seeded with murine ovarian cells, which adhered, formed follicle‐like structures, and produced E2 in vitro. When transplanted under the kidney capsule of immunocompetent and prepubertal mice, the grafts restored E2 and inhibin A levels, and induced vaginal opening in most animals—markers of puberty onset. Histological analysis confirmed cell survival, hormone production, and follicle‐like structures, with minimal immune response. Immunohistochemistry confirmed the presence of key protein ovarian markers, including FOXL2, alpha‐inhibin, and CYP17, indicating the maintenance of cell identity and function on the scaffold (Figure 6e,f).

**FIGURE 7:**
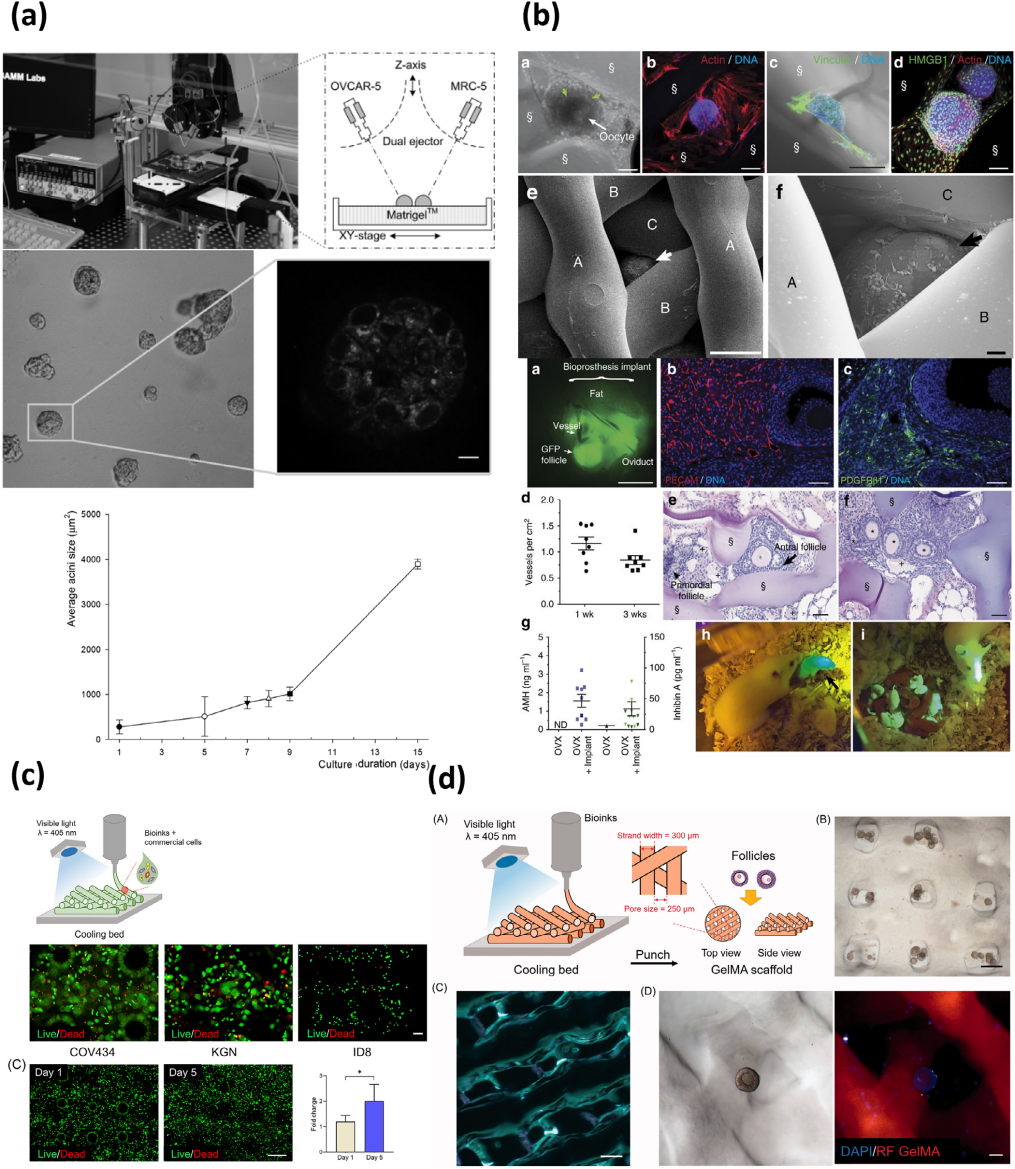
Cell seeding after scaffold bioprinting. (a) Cell patterning platform based on droplet bioprinting of ovarian cancer cells and normal fibroblasts. After bioprinting, cells formed acini structures on a Matrigel substrate. Adapted from Reference [84], © 2011 with permission from Wiley‐VCH. (b) Bioprinting of microporous scaffolds having different pore geometry (30°, 60°, and 90°), on which mice follicles were seeded. After implanting in mice, scaffolds displayed restoration of organ function. Adapted from Reference [85], this article is distributed under a Creative Commons Attribution (CC‐BY) license. (c) Extrusion‐based bioprinting of artificial ovaries by using commercial cells (c), and by seeding follicles on bioprinted scaffolds (d). Adapted from Reference [50], this article is distributed under a Creative Commons Attribution (CC‐BY) license.

A significant study exploiting a bioprinted scaffold on which cells were subsequently seeded was carried out by Xu et al. who developed an innovative 3D bioprinting strategy to resemble a complex model of ovarian cancer.^84^ In their study, they employed a droplet‐based cell printing platform capable of precisely depositing ovarian cancer cells (OVCAR‐5) and fibroblasts (MRC‐5) on a Matrigel substrate. The system exploited dual ejectors to pattern nanoliter‐sized cell‐laden droplets with exceptional accuracy (<3.5% positional error), while maintaining high cell viability (90.1% and 93% for OVCAR‐5 and MRC‐5 respectively). By adjusting the initial cell concentration, they were able not only to control the number of cells per droplet, but also the cell–cell distance, a crucial factor for modeling tumor‐stroma interactions. After printing, the cancer cells self‐organized into 3D acini that closely resembled ovarian cancer micronodules found in vivo and in earlier macro‐scale models (Figure 7a). These acini grew over time, showing heterogeneous size distributions due to proliferation and fusion events—mirroring biological behavior observed in patient‐derived tumors. The platform was cocultured with fibroblasts, allowing studies on tumor–stromal crosstalk. The formation of a reliable ovarian cancer demonstrates the feasibility of using bioprinting methods to generate reproducible and biologically relevant 3D cancer models, paving the way for high‐throughput drug screening and mechanistic studies of cancer progression.

A different approach was adopted by Laronda et al., who developed a 3D bioprinted prosthetic ovary for reproductive bioengineering.^85^ They focused on addressing the specific clinical need to restore ovarian function in patients, especially pediatric cancer survivors, whose fertility and hormone production are compromised due to aggressive treatments. In their work, they employed an extrusion 3D bioprinting strategy, resulting in a gelatin scaffold having a pore size of 350 μm large enough to accommodate both secondary follicles and aggregates of immature follicles. Resulting structures exhibited physiological mechanical features, showing a compressive elastic modulus of 16.84 kPa that closely matches native ovarian tissue stiffness.^92^ Gelatin was maintained at 30°C during printing in order to preserve it in a partially crosslinked state. At this temperature, the storage modulus (*G*′ ≈36 Pa) is sufficiently low to enable extrusion, yet upon further cooling (17°C, *G*′ ≈5400 Pa) the modulus increases nearly two orders of magnitude, ensuring mechanical robustness after deposition. This thermoreversible sol–gel transition, which occurs around 33°C when *G*′ ≈*G*″, reflects the progressive formation of triple helices and physical crosslinks as the temperature decreases, ultimately leading to a fully crosslinked gel at ~17°C. On the scaffold, they seeded murine follicles after removal from 16‐day‐old CD‐1 strain female mice. By adjusting the angle between printed layers (30°, 60°, or 90°), they explored how pore geometry impacts follicle viability, shape preservation, and function. Their results demonstrated that follicles survive and function optimally in scaffolds with 30° and 60° angles, where they maintain multiple contact points with struts, preserving their natural spherical architecture, critical for oocyte–granulosa cell communication (Figure 7b). Follicle‐seeded scaffolds were then implanted into sterilized mice. The implants became vascularized, produced hormones such as inhibin A and successfully integrated into the host's reproductive axis, culminating in progesterone‐mediated lactation and the birth of reproductively competent offspring. This study represents a significant advancement in the field of reproductive tissue engineering, offering a compelling proof of concept for functional ovarian restoration via 3D bioprinting. While further refinement is needed to scale the technology for human application, this study lays a strong foundation for future efforts aimed at developing clinically relevant, bioengineered ovarian grafts.

A critical aspect related to 3D bioprinting of the ovary was explored by Wu et al.^50^ In their study, they produced structures capable of resembling the ovary. As with Laronda et al.'s work,^85^ the aim of this study was to create a prosthetic ovary capable of supporting follicle growth and maturation, suitable for regenerative therapies targeting female reproductive and endocrine disorders. Initially, they investigated the efficiency of different bioinks, such as GelMA, alginate, and a GelMA–alginate blend. GelMA was selected as the optimal candidate due to its superior printability, shape fidelity, and mechanical robustness. They performed a cell‐laden extrusion 3D bioprinting of commercial tumor cell lines (COV434, KGN, and ID8), and observed high cell viability and low cytotoxicity, indicating the high feasibility of this approach. However, when attempting to replicate this strategy with primary ovarian somatic cells derived from mice, the results were slightly different. The extrusion process triggered massive cell death, likely due to shear stress and low‐temperature exposure, revealing widespread apoptosis. Despite this limitation, the authors succeed in demonstrating that exogenous ovarian follicles seeded on GelMA scaffolds (i.e., without bioprinting them within the grid) can survive, grow, and mature in vitro (Figure 7c). The scaffolds supported follicular development up to metaphase II oocytes, a milestone that suggests real potential for future therapeutic use. Interestingly, the architecture of the scaffold—specifically the grid angle—affected follicle distribution and survival, with 60° structures performing better than 90° ones. Their findings highlight the crucial limit of the immediate translational value of direct cell‐laden bioprinting, but offers an important technical insight, indicating the need to develop different printing strategies depending on the cell type and final goal to be achieved.

## 4 CELL‐LADEN SCAFFOLDS

Cell‐laden bioinks can potentially give several advantages such as homogeneous cell distribution throughout the structure and better spatial control, thus enabling the creation of more complex and physiologically relevant tissue‐like structures.^93^, ^94^

**FIGURE 8:**
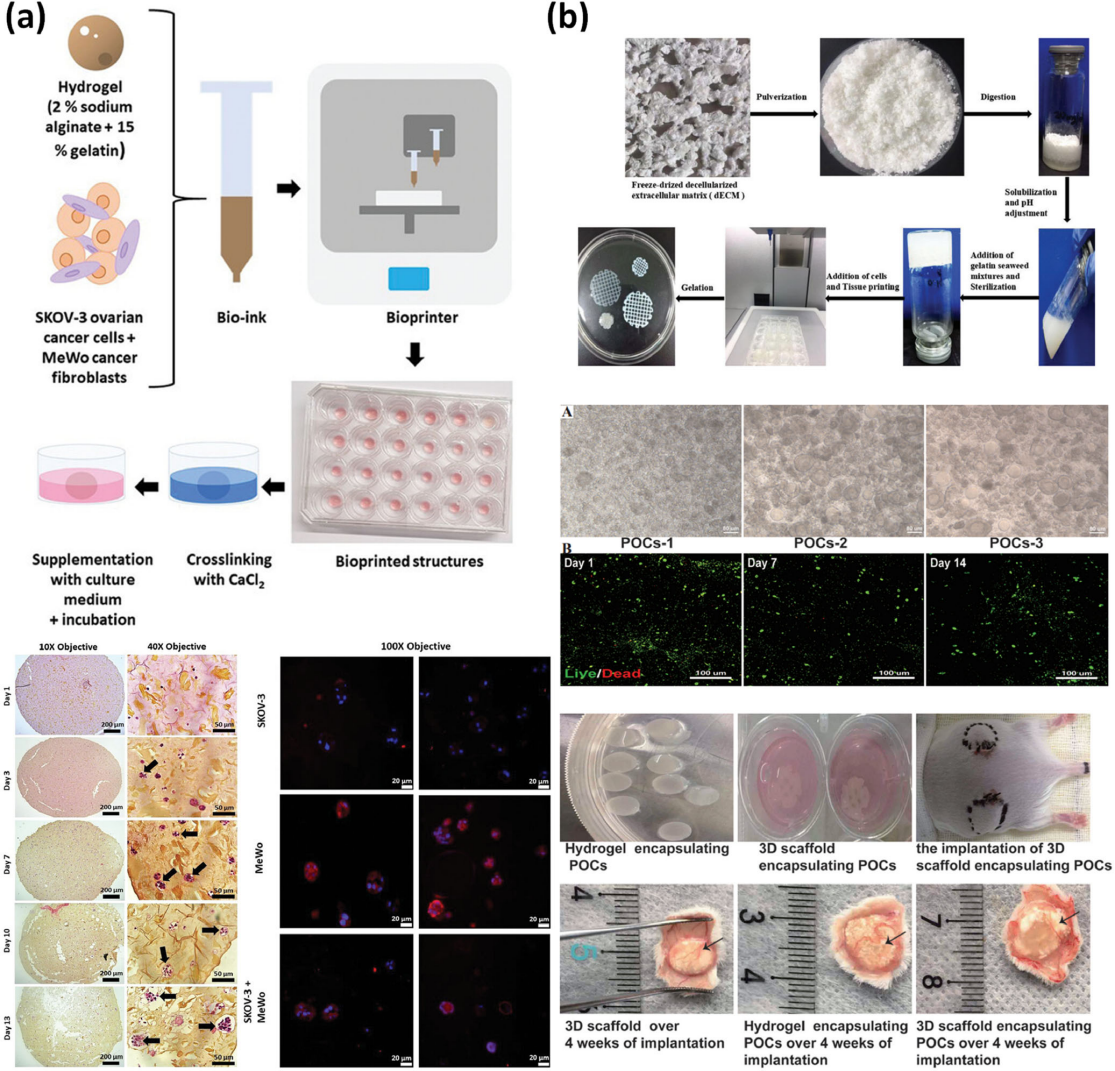
Cell‐laden bioprinting. Extrusion‐based bioprinting of ovarian cancer cells and cancer‐associated fibroblasts. (a) Cells formed spheroid‐like aggregates, resembling in vivo conditions. Adapted from Reference [87], © 2023 with permission from Wiley‐VCH. (b) Extrusion‐based bioprinting of ovarian cells within an alginate–gelatin bioink having decellularized porcine extracellular matrix. Adapted from Reference [86], this article is distributed under a Creative Commons Attribution (CC‐BY) license.

Baka and colleagues aimed at developing a 3D model of ovarian cancer, made of two commercial cell lines: SKOV‐3, an ovarian adenocarcinoma‐derived cell line and MeWo, working as cancer‐associated fibroblasts.^87^ The final shape of their bioprinted model, produced via an extrusion‐based technique, was a cylinder having a 7 mm diameter and a 1.5 mm thickness. The bioink used for this model was a mixture of sodium alginate and gelatin. Those polymers were initially mixed at different percentages, and the best was selected based on the printability of a square grid of 10 × 10 × 1 mm^3^. Once the mixture was selected, cells were printed at a final concentration of 1 × 10^6^ cells/mL, and biological parameters such as proliferation and viability were observed in a time span of 7 days (Figure 8a). Importantly, authors noticed a tendency of ovarian cancer cells to self‐assemble into spheroid‐like aggregates, resembling their behavior in vivo. Cancer‐associated fibroblasts were also observed migrating toward tumor cells, ultimately surrounding them and mirroring stromal encapsulation processes observed in metastatic ovarian cancer. Histological analysis performed on the bioprinted models depicted a high resemblance of ovarian cancer and its surrounding microenvironment. By culturing patient‐derived ovarian cancer cells from biopsies along with cancer‐associated fibroblasts, this work could serve as an effective tool for creating personalized tumor models that reflect inter‐patient tumor heterogeneity.

In contrast, Zheng et al. proposed a bioengineered ovarian construct that exploits the high biological fidelity of dECM, with fine precision warranted by extrusion‐based bioprinting.^86^ In their work, they obtained a dECM derived from porcine ovaries via decellularization. The dECM powder was then solubilized and mixed with a solution containing sodium alginate and gelatin (3% and 15% w/v, respectively). Primary ovarian cells were isolated from 4‐week‐old female Kunming mice, and mixed with the bioink containing dECM at a concentration of 1 × 10^6^ cells/mL. Bioprinting was performed in the shape of a porous circular grid, where the printed scaffolds displayed an average pore diameter of 75.58 μm. The structural characterization of the bioink revealed a typical collagen triple‐helical conformation, with thermal denaturation occurring between 45 and 70°C, thereby confirming the structural stability of the construct under physiological conditions. Rheological measurements showed that both the dECM solution and the composite bioink displayed solid‐like properties, with storage moduli G′ higher than loss moduli *G*″. Importantly, the *G*′ value of the crosslinked bioink (~2.4 kPa) was significantly higher than that of the uncrosslinked bioink (~0.7 kPa) and the dECM solution (~0.2 kPa), indicating enhanced mechanical stability upon crosslinking. The ovarian scaffold was cultured for 2 weeks and monitored in terms of viability and proliferation (Figure 8b). After ovarian growth, scaffolds were implanted into mice grafts. Authors observed high cell proliferation, survival and, importantly, significant angiogenesis. These results are in apparent contrast with the ones achieved by Laronda et al.,^91^ which needed to seed ovarian follicles after scaffold bioprinting due to poor viability of primary cells. However, the presence of dECM could play a key role in boosting and preserving cell viability. Sex hormone levels (E2, FSH, and P) in treated mice were restored close to physiological levels. Their hormonal and histological data indicate that the printed constructs successfully recreated aspects of ovarian function, including expression of ER‐α, FSHR, and inhibin‐α. This work could allow ovarian failure correction through 3D bioprinting of a primary cell‐laden functional model.

**FIGURE 9:**
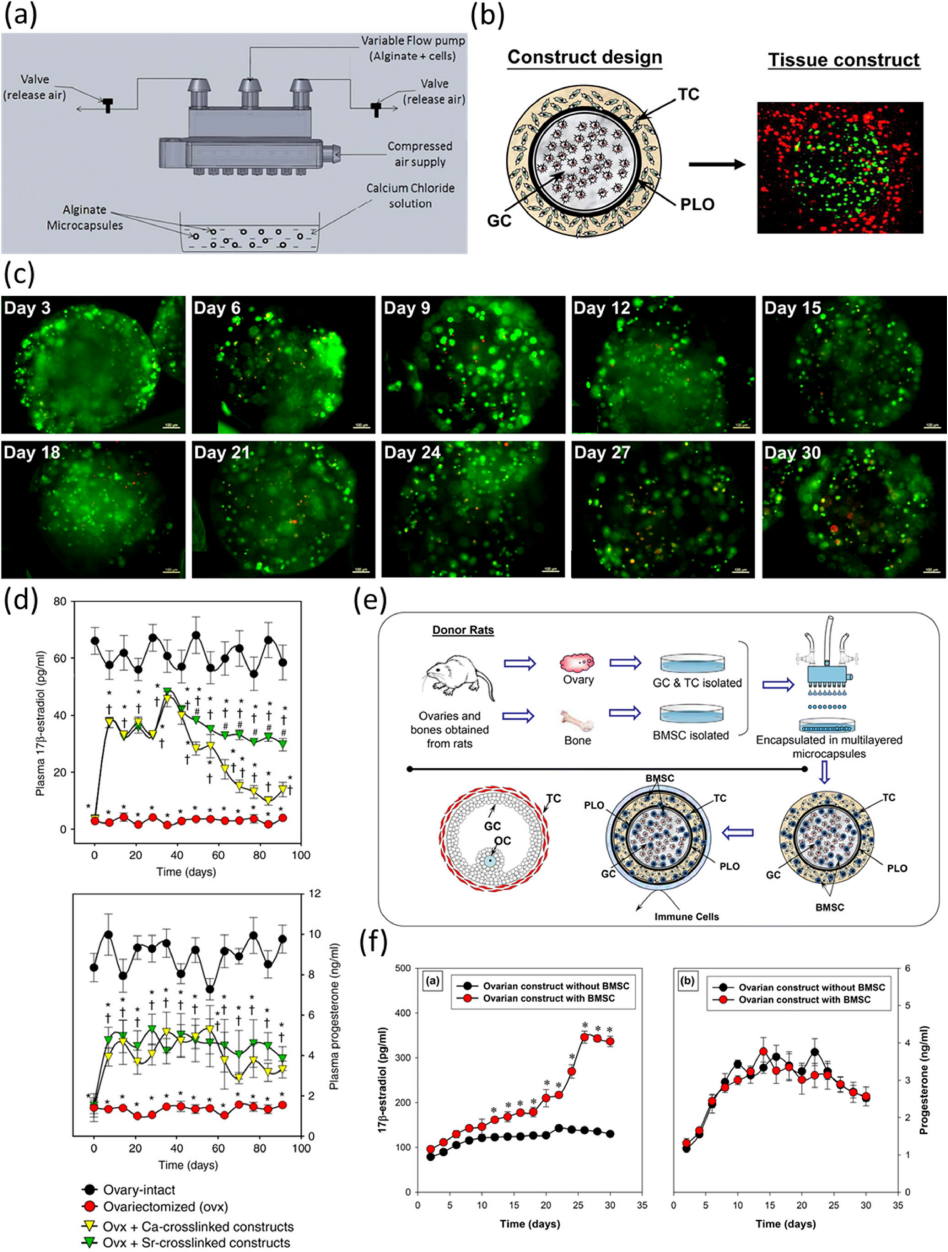
Microencapsulation of theca and granulosa cells. (a) Scheme of the encapsulation system through microfluidic device. Reproduced from Reference [95] © 2012, Springer. (b) Multilayered microcapsule mimicking native follicular structure with granulosa cells labeled in green and theca cells labeled in red and (c) corresponding live/dead staining over time. Reproduced from Reference [96] © 2012, Elsevier Ltd. (d) Plasma hormone levels in sham and ovx rats treated with constructs crosslinked with Sr++ or Ca++. Reproduced from Reference [97] © 2017, The Author(s). (e) Scheme of the biomimetic ovarian constructs incapsulating BMSC. (f) In vitro estrogen secretion in the presence/absence of BMSCs. Reproduced from Reference [98] © 2019, Biomedical Engineering Society. GC, granulosa cells; TC, theca cells.

Another relevant technology employed to obtain cell‐laden scaffolds resembling ovary involves the use of microfluidics. Recent advancements in microencapsulation and tissue engineering have significantly enhanced the potential of cell‐based therapies, particularly in the context of endocrine function restoration. Traditional microencapsulation systems, such as air‐syringe pump and electrostatic bead generators, have been constrained by limited throughput, posing challenges for clinical‐scale applications. To overcome this, a novel microfluidic device featuring a multi‐nozzle design has been developed (Figure 9a), enabling high‐throughput encapsulation of cells and proteins in alginate‐based hydrogel microbeads, thereby improving scalability and quality control^95^ This technological improvement provides a robust platform for therapeutic applications, including hormone replacement therapies (HRT). For instance, encapsulation of granulosa and theca cells in multilayer alginate microcapsules (Figure 9b,c) has been shown to better mimic the native follicular architecture, resulting in sustained and elevated E2 secretion over extended in vitro culture.^96^ Building on this, 3D bioengineered ovarian constructs crosslinked with divalent cations such as calcium or strontium have demonstrated the ability to restore hormone levels and improve physiological deficits in ovariectomized rat models (Figure 9d), offering a safer alternative to pharmacological HRT.^97^ Importantly, the inclusion of bone marrow‐derived mesenchymal stem cells (BMSCs) within these constructs further enhances estrogen production (Figure 9e,f), likely via paracrine signaling and local aromatase activity, leading to improved systemic hormone regulation and tissue‐specific outcomes such as prevention of uterine atrophy.^98^

## 5 CONCLUSIONS AND FUTURE CHALLENGES

3D bioprinting holds substantial promise for medical applications, particularly in addressing organ failure and tissue damage. In this review, we have highlighted the significant advances in the development of 3D bioprinted ovaries. Given that the ovary is both a reproductive and endocrine organ, these innovations necessitate careful ethical and legislative considerations.^99^, ^100^

As of now, the estimated cost of printed organs remains high, leading to concerns that access may become a privilege exacerbating ethical issues related to social stratification.^101^ Additionally, there are concerns regarding the intended use of 3D bioprinted organs, questioning whether transforming them into artificial organs addresses critical illnesses or serves solely as a rejuvenation technology. If this technology progresses into commercialization in the future, ethical and regulatory boundaries between clinical applications and human enhancement must be clearly defined.^99^, ^101^

Other concerns are related to the cell source and its ownership and utilization. In the cases of allogeneic cell transplantation, the donor's confidentiality and informed consent deserve significant attention. In addition, the ownership of the 3D‐printed product, and the authorization of the donor, recipients of the organ, the researcher and the company that intend to utilize it is a topic that cannot be ignored.^29^, ^102^

Since the ovary is a reproductive organ, biofabrication is not only related to the patient's health but also to the well‐being of future offspring.^99^ Although 3D ovarian bioprinting mainly benefits patients that suffer from irretrievable loss of reproductive and ovarian endocrine function, the ex vivo manipulations with ovarian follicles might lead to genetic and epigenetic changes in the oocyte, which directly affect the offspring's health.^99^ Furthermore, in some settings, such as in BRCA‐mutated patients, safety must be given particular attention, as it could be hypothesized that the implantation of bioprinted ovaries might induce the risk of tumor degeneration in cells that are “per se” predisposed. Also, potential effects that an artificial ovary might have on a patient's physical and psychological welfare must be taken into account.^99^

The possibility of generating oocytes from fibroblasts via iPSCs represents a significant scientific advancement but also raises important ethical concerns.^103^ This approach could revolutionize fertility treatments by offering new solutions to infertility, particularly for individuals unable to produce functional oocytes. However, one major ethical issue revolves around the use of these lab‐generated oocytes in human reproduction. If the technology advances to the point where viable human embryos could be created, questions arise about the potential for genetic manipulation, the creation of embryos for research purposes, or even the selection of specific genetic traits. The scientific community, ethicists, and regulatory bodies must work together to create frameworks that ensure the responsible use of this technology. Ethical guidelines should prioritize patient welfare, avoid harm, and safeguard against potential misuse in both clinical and research settings. Therefore, given the multitude of issues to address, achieving consensus within society still has a long way to go. It should also be acknowledged that ovarian tissue transplantation itself has shown long‐term benefits, with the potential to restore natural fertility and endocrine function, delay premature menopause, and improve quality of life, sometimes for more than a decade after transplantation. These encouraging outcomes highlight the promise of ovarian bioengineering but also emphasize the importance of carefully evaluating both the durability of graft function and the broader implications for patient welfare.^104^ While ovarian tissue transplantation might enable the restoration of endocrine function and, in some cases, fertility, follicles within transplanted ovarian tissue may undergo accelerated activation, leading to premature depletion of the follicle pool and subsequent atresia within months post‐transplantation. This phenomenon, often attributed to disruption of the ovarian microenvironment and ischemic injury following grafting, poses a major challenge for the sustained functionality of transplanted ovarian tissue. Addressing these limitations remains a crucial goal for improving transplantation outcomes and highlights the potential role of bioengineering strategies, such as 3D‐printed scaffolds, in better supporting follicle survival and maturation.

Also, since bioprinted products, with their complex cell sources and bioink composition, can be classified as biologic or medical devices, they do not fit neatly into existing regulatory frameworks. Current legislation fails to adequately address the ethical implications of artificially creating human body parts.^105^ Consequently, these products necessitate more tailored regulations and laws governing clinical trials and commercialization.^101^

## AUTHOR CONTRIBUTIONS

**CEH** and **MM**: Conceptualization, literature review, writing—original draft. **GP**, **AM**, **VF**, **CP**, **GA**, and **AA**: Visualization, data curation, writing—review and editing. **VP**, **CM**, **RE**, **CN**, **MDS**, and **MP**: Resources, supervision, project administration, funding acquisition, and writing—review and editing. All authors have read and approved the final version of the manuscript.

## CONFLICT OF INTEREST STATEMENT

This article is a narrative review and does not involve any studies with human participants or animals performed by any of the authors. The authors declare no conflicts of interest relevant to this work. Data sharing is not applicable to this article as no new data were created or analyzed in this study.

## Data Availability

The data that support the findings of this study are available on request from the corresponding author. The data are not publicly available due to privacy or ethical restrictions.
